# Elements of Pathology and Therapeutics, &c.

**Published:** 1825-07-01

**Authors:** 


*Elements of Pathology and Therapeutics, being the Outlines of a Work intended to ascertain the Nature, Causes, and most efficacious Modes of Prevention and Cure of the greater number of Diseases incidental to the Human Frame; illustrated by numerous Cases and Dissections.* By CALEB HILLIER PARRY, M.D. F.R.S. &c. Vol. I. General Pathology. Second Edition. 8vo. pp. 374, with an Appendix, pp. 46. London, January, 1825.

WE were among the first to hail with approbation the original edition of this work, and none of his warmest friends could have, more sincerely than ourselves, deplored the lingering but fatal stroke which deprived the world of its author. Dr. Parry's Elements of Pathology, though not without defects of a most important kind, will stand an honorable monument of British medicine, as long as the science itself continues to be cultivated. We twice brought it before our readers in very extended analyses, and therefore we have only to notice in this article the Appendix of new matter—which, by the way, Dr. Parry ought to have published in a separate form for the benefit of the holders of the first edition, since very little alteration is here made in the text of the original work. It is particularly with the view of supplying this omission of the present editor, that we shall dedicate a space to the Appendix, somewhat out of proportion to its actual magnitude, though not more than commensurate with its real value.

Of the elder Parry's proposed second volume, this Appendix was the only portion completed, when an assault of apoplexy put a period to that lamented physician's professional career—though life was protracted for some years afterwards. It is also remarkable that the last page of it was finished on the day preceding the melancholy event abovementioned! such is the frail tenure of man's existence—such the uncertainty of his mundane prospects!

The portion of work then, to which we have to draw the attention of our readers, forms, as it were, the preliminary dissertation to the therapeutic or second volume, which is promised by the son of the deceased author. It is more etiological than any part of the first volume, as far as remote causes are concerned. It is on "*the effects of habits in creating predisposition to disease*," and is divided or arranged in the same aphoristic manner as the preceding part of the publication. This method has its disadvantages—but is not without its advantages also. The matter, however, is valuable, whatever be the form in which it is conveyed.

In the preceding part of the work, Dr. Parry had endeavoured to establish certain general principles regarding the majority of diseases, and to shew that, *in all*, there was one condition, either cognizable by the senses, or admissible by induction; namely, a degree of fullness or momentum of the blood, excessive with regard to the usual state of the vessels of the part, or other circumstances of the system. He now comes to an inquiry relative to the more remote causes, whether constituting predisposition or excitement. And first it is to be inquired why man, in civilised life, is more prone to disease than man in a state of Nature.

1. A primary cause may be traced to that disposition, (exhibited from the cradle to the grave) for gratifying every want, and removing every present suffering. This disposition, it is true is inherent in our very nature, and was no doubt implanted there for the preservation of existence; but then, in civilised life, it is carried to a degree of perversion that proves the source of the principal physical evils which afflict mankind.

2. Education among civilised nations is more instrumental in the production *even of moral evil*, than a state of ruder nature.

"In the former state, the wants of infancy and childhood are satisfied as soon as expressed, and even anticipated and prevented. A similar conduct is observed with regard to those slight sufferings which are incidental to the frail state of humanity. Under both these situations, instead of being taught patience by reasonable denial, it usually happens that the child is gratified precisely in proportion to the violence of importunity; or else bribed into acquiescence by some commutation of sensual indulgence.

"Thus he learns to consider present enjoyment as the end of his existence; and, concluding that the world was made for his pleasure, is averse to the torture of instruction, and hates those who contradict, or oppose him." 7.

To this criminal indulgence of innate propensities during childhood, may chiefly (in our author's opinion) be traced those vicious habits which, growing with our growth, prove the bane of all social virtues, and unfit us alike for present and future happiness!

In the lower orders of society, although the child be brought up more hardily, he soon discovers that he can disobey with impunity, and that, in spite of repeated positive denials, he can always, by querulousness, passion, or persevering importunity, gain his purpose ultimately.

"In no rank of life is there, in general, much choice as to the means, provided the end be obtained. If the child cry, it must be quieted. If benefit be to be procured, at the expense of inconvenience, however trifling, either the good must be absolutely relinquished, or acquiescence must be gained by some assurance or promise, which, the very next moment, the child discovers to be a falsehood." 8.

These facts, which medical men have daily opportunities of verifying, must lead us to conclude that the habits of civilised society place the dawn of human life in a worse situation, with regard to the common principles of virtue, than those of the untutored savages.

It is but too true that, in the conduct of the parent and nurse may be found the cause of this evil. They act towards the child in conformity with their own habits. Unaccustomed to regulate their own conduct beyond the feelings of the present moment, they apply the same rule to their little charge. They see that he is annoyed by the exertion of due authority, and his expressions of this annoyance are very disagreeable to them. They are, therefore, anxious to accomplish their own immediate ease—and do not discover, till too late, that they have defeated their own purpose, and rendered inveterate those very evils which they strove to shun! But now for the physical effects of this conduct on health.

"Human life consists in a continued series of irritations; and Providence has so ordered, that scarcely any earthly good is to be acquired without labour and suffering. As also, on one hand, the good itself is enhanced by the difficulty with which it is attained; so, on the other hand, there are few pains, whether of body or mind, which are not, within certain limits, alleviated by continuance or repetition. Hence, no one, probably, enjoys true pleasure, but he who has experienced pain; and moderate evils are scarcely felt by those, who have been habituated to greater. On the contrary, men who are nurtured in habits of self-indulgence, not only bear with impatience those inconveniences, which are common to the rest of mankind, but possess an acuteness of perception, which converts indifferent impressions into actual sufferings." 10.

The predisposition being thus created, every thing which can contribute to the eventual torrent of the unhappy being through after life, is, on principle, superadded. To this end, *exercise*, which formed the chief pleasure of youth, is disregarded in adult age. Hence, in males, we find dyspepsia, head-ache, and various affections of the brain—and, at a more advanced period, gout, dropsy, and the host of what are termed bilious complaints.

With the exception of gout, these evils fall still heavier on the female sex, who, from the rules of society, are very early subjected to physical restraint, being long confined in sitting postures under governesses—and in the intervals of study being compelled to relinquish all those exercises in which boys freely indulge.

"Nay, there are certain muscles, the use of which fashion imperiously commands females to forego through life. Under the pretext of obtaining support to the trunk of the body, they are from their childhood cased up in stays, which prevent any flexion of the back bone. They can bend themselves only by the neck and hip joints, while the whole spine, from the neck downwards, is rigid and immoveable. Hence the muscles of the trunk, from disuse, become weak; and the ignorant parent or governess, finding herself, at adult age, incapable of keeping her own body erect without stays, fancies that this machinery is absolutely necessary to supply the defective work of Providence, in the construction of the child." 12.

Such folly is visited by its proper punishment. To this cause, and the general want of other bodily exertion, is, in a great degree, attributable that disposition to distortion, so common among European females of a certain rank in life—but which is almost totally wanting among negroes or other uncultivated nations.

"Of this mechanical durance there is also another effect, hitherto, I believe, overlooked by pathologists. The chief muscle of respiration is the diaphragm; which, in healthy men, is alone used during rest, especially in the horizontal posture. In women, on the contrary, the stays, compressing the upper part of the abdomen, prevent the descent of the diaphragm, and therefore compel them to inspire by the thoracic muscles only. Hence while females sit or stand, even at rest, the breasts rise and fall during respiration; and such is their habitual disuse of the diaphragm, that even when unrestrained by cloathing, and in the horizontal posture, they still breathe chiefly by the intercostal muscles." 13.

To the above observation, which is very just, may be added, the effect of these abdominal compresses on the intestines themselves, as undoubtedly checking their peristaltic motions, and thus contributing to that state of constipation so generally prevalent among females. But a still worse consequence may be, as Dr. Parry suggests, entailed on the lungs, by this unnatural check to the play of the diaphragm. The subserviency of free respiration to the growth, health, and strength of the animal frame, is well known; and we can, therefore, readily understand how much the want of exercise, aided by the mechanical impediment above alluded to, must tend to counteract those blessings, and to prevent or destroy that beauty which, without them, cannot exist. Chlorosis, our author thinks, is not seldom a consequence of this tight lacing and muscular inactivity—an idea supported by the almost absolute certainty with which chlorosis can be cured, without the aid of medicine, by a due degree of muscular exercise in the open air. Scrofula, and its consequence, tubercular phthisis, are, in all probability, greatly increased by the want of regular exercise in the air. The following Philippic against passive and even *horse-exercise*, is rather too severe, especially as regards the *latter*. Dr. Parry's observations, as far as they apply to *carriage exercise*, are, we fear, but too well founded.

"Of the species of self-indulgence thus described, the natural offspring is the habitual use of Gestation, whether on horseback or in carriages. With regard to riding on horseback, it is usually a mere apology for the want of that exercise, which Providence evidently intended that man should take by means of his own limbs, and not those of another animal. Accordingly we find that, exclusively of the positive diseases which spring from this mode of gestation when violent, those who trust to its more moderate use, and, more especially, those who substitute it for accustomed bodily labour, are at least as subject to dyspepsia, gout, dropsy, hæmorrhage, the whole train of nervous affections, mania, hysteria, epilepsy, paralysis, and apoplexy, as those who lead the most indolent lives. Many persons indeed assert their inability to walk to any extent, or to employ any other active exertion. It heats, it fatigues, it pains them; it produces a thousand real or imaginary inconveniences; whereas the exercise of riding is in no degree fatiguing, and, therefore, must be infinitely more beneficial. This very argument is an absolute dereliction of the point which it wishes to establish; since it proves, first, that the exercise of certain muscles gives strength to those muscles, while it leaves in a state of debility others, which are habitually unexerted; and, secondly, that the indisposition to incur present inconvenience by such exertions of new muscles, implies that state of mind, already mentioned as being weak, and worthy of reprobation." 15.

Of the power of exercise to strengthen muscular parts, we have daily experience, not only in man, but in the inferior animals. "We might otherwise, says Dr. P. expect to see, in Europe, greyhounds, trained for coursing, astride of hunters; and, in India, horses fitted for racing, by being trotted on the backs of elephants." The following is truth itself.

"Whoever pleads mere fatigue, or a temporary diminution of ease, as a reason for shunning muscular exercise, and waits for that exercise, till his strength shall have been previously improved, inverts the true order of nature, by which, conformably to a well-known proverb, it appears, that increased muscular power is the effect, and not the cause, of increased muscular exertion." 16.

Dr. Parry reverts again to the mistaken notions (as he supposes) entertained of horse-exercise. Under such exercise, it is not uncommon for the feet to get cold—the face to flush—and the lower extremities to waste, in comparison with other parts of the body. Under this habit also, the heart, like voluntary muscles, is thrown into inordinate actions by the slightest irritation, whether from exercise or mental emotion; the due balance of the circulation is thus disturbed—the blood undergoes a defective decarbonization—and the secretions and excretions are either diminished on one hand, or more or less extensively increased on the other—or, what is still more common, they are vitiated as to their quality. The maladies to which these habits lead are of a most serious and extensive nature, comprehending those of the heart, lungs, alimentary canal, and liver—the whole train of nervous affections, including excessive determinations of blood to the head—all inflammatory diseases, together with the various effusions, the consequences of these several states.

If these consequences result from habits of horse-exercise—they must be still more certainly produced by gestation in carriages. These observations do not, of course, apply to the cases of those persons who, either from organic infirmity of their limbs, or from the debility consequent on acute diseases, are, at the same time, incapable of moving their bodies by means of their own muscles.

"The indolence to which I have adverted, is peculiarly injurious when conjoined with full meals, especially of animal food. To such excess we are, however, habitually stimulated by every variety of taste produced by the arts of cookery, by the admixture of condiments, and by the interposition of Alcohol in its different forms. This habit of indulgence in the pleasures of the table, under various modifications, especially occurs to persons rather beyond the middle periods of life; to men who have become affluent in laborious occupations, and to females who have been pampered during frequent pregnancies. From the combination of indolence and gluttony arises a degree of obesity, as offensive, as it is destructive of the little mental and bodily power, which the prior habits themselves would otherwise have permitted to remain." 19.

The use of wine and alcohol next comes in for Dr. Parry's rigid censure. He observes, and we fear with justice, that few persons would habitually accustom themselves to wine or spirits, as a part of their daily meals, were they not temporarily relieved from certain degrees of mental or bodily torpor, which they have not the fortitude to sustain, or the patience to relieve, by slower, but more effectual means.

"In reality, the extent of this habit among men in certain ranks of life, is enormous; and persons who would with disdain repel the imputation of drunkenness, are, nevertheless, satisfied to continue the use of such liquors, in any quantity which may serve, for a time, to raise their bodily and mental power above their natural and healthy level, provided they stop short of absolute inebriation. Nothing, however, can be more erroneous than such a conclusion; since it is true, in fact, that those persons who act on this principle are subject, at least equally with habitual drunkards, to the fatal effects of such practices. Of this assertion it would be easy to accumulate proofs from the examples of those who have acted the most conspicuous parts on the public theatre of life." 20.

Our author is compelled, though with regret, to attribute to the medical profession itself much of that perseverance in these delusive modes of exercise, and in the habitual use of stimulating fluids, which have here been reprobated—practices to which mankind is sufficiently prone, without any sanction from medical authority.

To the same overwhelming authority of fashion, persons in the middle and higher ranks of life are indebted for an almost total inversion of the periods which Providence, with a strong and commanding voice, has appropriated to the different purposes of exercises and rest.

"Late hours naturally lead to fever, and proportionate lassitude, and it is hardly probable that he who, from whatever cause, has watched through the greater part of the night, will feel himself disposed, or able, to pursue the means of bodily and mental health through the ensuing day." 21.

Even the effect of *light* upon the animal frame, has not, Dr. P. thinks, been considered according to its real importance. Another evil, in civilized life, is the habitual exposure to the heat of large fires, and the indiscriminate use of flannel garments next the skin, both of which tend to produce excessive action of the heart, and to make the frame morbidly susceptible of the action of cold during that exercise which is essential to health.

"In a former part of this work it has been observed that various animals dependent on man, and accustomed to indolence and similar indulgences, acquire a disposition to like diseases. Thus singing-birds and lap-dogs, which are confined and highly fed are subject to the whole train of nervous affections; as palpitation of the heart, breathlessness on slight motion, hysteria, convulsions, epilepsy, hemiplegia, and apoplexy. So of various diseases in other domesticated animals." 22.

These general facts shew, beyond all question, that the predisposition to the worst maladies which flesh is heir to, grows out of the habits of civilized life. To the early desuetude of restraint, and the habits of self-gratification, we may also attribute many of those intemperate indulgences of natural appetites which predispose to and excite various maladies.

"Inde apud mares oritur Veneris cultus præcox et effrænus; quo nihil mentem magis infirmat, nihil corporis vires magis frangit, nihil articulorum, ventriculi, cordis, cerebri morbis, virum magis obnoxium reddit." 23.

"PREDISPOSITION FROM HABITS, A CAUSE OF INCREASED DETERMINATION."

If it be true, Dr. Parry observes, that the different circumstances abovementioned give occasion to the more ready production of the local diseases, and if it be also true that a main and essential link in the phenomena of these diseases, is an excessive determination of blood to the parts so affected, it must follow that these predisposing causes tend to the production of that increased determination. This state must be the joint product of the condition of the part, and the exciting cause acting on it. Thus, under a strong predisposition, a slight exciting cause may be sufficient, and conversely, under a slight predisposition, a strong exciting cause is requisite.

On a former occasion, our author has endeavoured to prove that the larger arteries have two powers, by which, when dilated to a certain degree, they can, within certain limits, contract themselves—the first, and least extensive, being the mechanical power of elasticity; and the second, and most extensive, being their tonic or vital power.

"I have also proved that, in health, those arteries are always in a forced state of dilatation with regard to both powers; so that when the distending cause, or the blood which they contain, is removed, both their elasticity and tonicity visibly act, and jointly tend to reduce the light of the vessel. Since, also, it appears that, as long as organic life continues, the tonic power of such vessels contracts them within the sphere of their elastic power, which, however, operates to re-expand them to a certain degree, after their organic life has ceased, we have reason to infer that this tonicity acts only in a mode similar to that of muscular parts, that is, by shortening the texture possessing it; or, in a hollow part, by reducing its area. Now, as there is reason to believe that capillary vessels, employed as conduits for carrying on the circulation, have powers similar to those of the larger arteries, though, perhaps, differing as to the proportion of those powers, we have no reason, either from analogy or actual observation, to conclude that, when unduly dilated, they owe this state to any other causes than either an increased force of dilatation within, or a defect either of tonicity or mechanical resistance. When, however, we observe the sudden changes which occur in the state of these vessels, so that not only the contact of a grain of sand with the conjunctiva shall almost instantly fill with blood the capillaries of that part: and, more especially, when we contemplate the instantaneous effects of certain mental emotions, in causing a rush of blood into the colourless vessels immediately under the skin, without our being able, in either case, to discover any adequate change in the movement of the heart; we feel ourselves compelled to deny, that such causes can act immediately on the elasticity of the vessel, and, on the contrary, are disposed to conclude, that the phænomenon is dependent on a change produced on the tonicity of the minute vessels of the part." 27.

We have introduced the foregoing extract, principally to shew what erroneous notions have been entertained of Dr. Parry's doctrines:—most of his opponents representing him as maintaining that the arteries of the human body are like so many "inert tubes," and have no other office or power than that of passing along the blood from the heart to the veins.

But, although this be the main part of their office, it is evident that they are endowed with other and very important powers, which are perpetually called into action by the various wants and conditions of the animal economy. But to resume the thread of the subject.

Under this view, the cause of predisposition in the vessels of a part is their defect of tonicity or vital contractility, which may exist in such a degree, as to admit of diseased conditions, even under the natural impetus of the blood.

"Why, from the causes which have been detailed, such a disposition to local defect of tonicity should take place, we can, in many respects, at best only plausibly infer. With regard to mental causes, we see a considerable degree of analogy between their action on the tonicity of vessels through the sensorium commune, and the operation of pressure on the brain, in producing paralytic relaxation of various muscles. There is, however, another analogy of a more obvious kind; which is the diminished contractility usually observable in muscles, after they have been long or violently stimulated to action. On this principle, we may reasonably explain the disposition to excessive determinations of blood, which is so apt to follow violent or long-continued increased impetus in certain parts, or the whole, of the sanguiferous system; such impetus having incapacitated the capillary vessels from that degree of vital contractility, which is necessary to the continuance of their healthy functions." 29.

Hence we find that persons who, during what is called health, have a preternaturally quick or strong pulse, are much more prone to all the diseases of excessive local determination than others, whose circulation is constitutionally more moderate.

"From what has been said it seems to follow that, whatever may be the causes of excitement, the state immediately constituting the disease in such cases, is the want of a due degree of tonicity in the capillaries immediately affected." 29.

It would be an interesting point to ascertain through what media the several causes of predisposition operate in increasing the general or local momentum of the blood. Our author thinks it is obvious that all of them, except indolence, immediately produce an increased action of the heart, and consequently, for the most part, an increased general momentum of blood—and, secondly, that indolence and full living produce a tendency to general plethora or fulness of blood, as is evinced by the pulse, venous distention, general heat, and, frequently, obesity.

The remainder of Dr. Parry's Appendix is taken up with physiological discussions on the circulation of the blood, and need not here be entered on. We shall look, with great anxiety, for the completion of Dr. Parry's work by his talented and ingenious son.

